# Gestational GenX Exposure Induces Maternal Hepatotoxicity by Disrupting the Lipid and Bile Acid Metabolism Distinguished from PFOA-Induced Pyroptosis

**DOI:** 10.3390/toxics13080617

**Published:** 2025-07-24

**Authors:** Jin-Jin Zhang, Yu-Kui Chen, Ya-Qi Chen, Qin-Yao Zhang, Yu Liu, Qi Wang, Xiao-Li Xie

**Affiliations:** 1 Guangdong Provincial Key Laboratory of Tropical Disease Research, Department of Toxicology, School of Public Health, Southern Medical University, No. 1838 North Guangzhou Road, Guangzhou 510515, China; zjj1881@smu.edu.cn (J.-J.Z.); cyk22020064@smu.edu.cn (Y.-K.C.); y22220102@smu.edu.cn (Y.-Q.C.); 15298213896@163.com (Q.-Y.Z.); 3180090110@smu.edu.cn (Y.L.); 2 Department of Forensic Pathology, School of Forensic Medicine, Southern Medical University, No. 1838 North Guangzhou Road, Guangzhou 510515, China

**Keywords:** GenX, PFOA, maternal, hepatotoxicity

## Abstract

Perfluorooctanoic acid (PFOA) and its replacement, GenX, are per- and polyfluoroalkyl substances (PFASs) widely used in industrial and consumer applications. Pregnant women are a vulnerable population to environmental pollutants. The maternal effects of GenX and PFOA exposure during pregnancy have not been fully elucidated. In this study, pregnant mice received daily oral doses of GenX (2 mg/kg/day), PFOA (1 mg/kg/day), or Milli-Q water (control) throughout gestation. Histopathological analyses revealed significant liver abnormalities in both exposure groups, including hepatocyte swelling, cellular disarray, eosinophilic degeneration, karyopyknosis, lipid vacuolation, and increased inflammatory responses. Through transcriptomics analyses, it was found that multiple metabolic and inflammatory pathways were enriched in both exposure groups. In the GenX group, overexpression of CYP4A, c-Myc, and Oatp2 proteins and decreased expression of EGFR and β-catenin in the liver suggested disruption of lipid and bile acid metabolism. In the PFOA group, significantly upregulated protein levels of NLRP3, GSDMD, caspase-1, IL-18, and IL-1β indicated hepatic pyroptosis. Despite these distinct pathways, both compounds triggered inflammatory cytokine release in the liver, consistent with the results of the transcriptomics analysis, suggesting shared mechanisms of inflammatory liver injury. Taken together, our findings provided novel insights into the hepatotoxicity mechanisms of GenX and PFOA exposure during pregnancy, underscoring the potential health risks associated with PFAS exposure.

## 1. Introduction

Pregnancy is a period of significant physiological transformation in the mother, influencing both maternal health and fetal development [1]. During gestation, maternal sensitivity to exogenous chemicals increases, allowing these substances to potentially cross the placental barrier and transfer from the mother to the fetus. This transfer may contribute to the developmental origins of health and disease (DOHaD), adversely affecting the long-term health of offspring [2,3]. The liver, as the primary organ for metabolism and detoxification, plays a critical role during pregnancy by maintaining maternal metabolic homeostasis and indirectly influencing fetal health [4,5]. Consequently, impaired liver function during pregnancy may adversely affect offspring development and health [6].

Per- and polyfluoroalkyl substances (PFASs) are a class of synthetic chemicals that have been extensively used in industrial applications and consumer products, such as textiles, firefighting foams, and nonstick products [7]. These compounds are ubiquitously detected in various environmental matrices, including surface water, soil, sediment, and biota [8,9,10,11]. Recognizing the environmental and health risks associated with these persistent compounds, the international community has taken regulatory action. For instance, perfluorooctanesulfonic acid (PFOS) and its related compounds were listed in Annex B of the Stockholm Convention in 2009 [12], perfluorooctanoic acid (PFOA) and its associated compounds were added to Annex A in 2019 [13], and perfluorohexanesulfonic acid (PFHxS) and its related compounds were included in Annex A in 2022 [14]. As long-chain PFASs have been gradually phased out, the production and environmental detection of short-chain alternatives have consequently increased. Studies have reported elevated concentrations of perfluorobutanoic acid (PFBA), perfluorobutane sulfonic acid (PFBS), and chlorinated polyfluorooctane sulfonate (F-53B) in surface waters near manufacturing facilities [15,16]. Similarly, GenX, an emerging replacement for PFOA, has been widely detected in surface waters around the world [17]. In light of the potential negative health effects of GenX on the environment and biota, the European Chemicals Agency (ECHA) added GenX to the list of substances of very high concern (SVHC) on 16 July 2019 [18].

Human exposure to PFOA and its alternative GenX occurs through various routes, with diet and drinking water being the primary sources [9,19]. Epidemiological studies have linked PFAS exposure to an increased incidence of gestational diabetes, childhood obesity, preeclampsia, and fetal growth restriction [20]. These adverse health outcomes may have significant implications for the long-term health of children. Research has demonstrated that both PFOA and GenX exhibit potential hepatotoxicity, inducing various liver pathological changes associated with the activation of inflammatory responses, initiation of apoptosis, and induction of autophagy processes [21]. Furthermore, exposure to these compounds may disrupt the intestinal barrier and trigger inflammatory pathways in the maternal liver [21]. Even at environmental concentrations found in drinking water, GenX can disrupt hepatic lipid metabolism by activating the peroxisome proliferator-activated receptor alpha (PPARα) signaling pathway, leading to metabolic disorders [22]. These findings suggest that GenX hepatotoxicity is closely associated with fatty acid transport, synthesis, and oxidation pathways, with PPARα playing a central role in these processes. Despite these advances in understanding the potential toxicity of PFOA and GenX, further investigations are needed to elucidate their specific effects on maternal liver function during pregnancy and the underlying mechanisms of action.

The present study investigated the maternal hepatic effects of gestational exposure to GenX or PFOA in mice. Using a C57BL/6J mouse model, pregnant mice were exposed to GenX (2 mg/kg body weight/day) or PFOA (1 mg/kg body weight/day) throughout gestation. The maternal hepatic effects and underlying molecular mechanisms were explored through transcriptomic analysis. By elucidating these mechanisms, our findings may provide a scientific foundation for understanding DOHaD related to PFAS exposure, which has significant implications for public health protection.

## 2. Materials and Methods

### 2.1. Chemicals and Reagents

PFOA (Ammonium Pentadecafluorooctanoate, CAS #3825-26-1, purity 98.4%) was purchased from Tokyo Chemical Industry Co., Ltd. (Tokyo, Japan). GenX (Ammonium Perfluoro (2-methyl-3-oxahexanoate), CAS #62037-80-3, purity 95%) was obtained from Toronto Research Chemicals (Vaughan, TO, Canada). Both compounds were dissolved in Milli-Q water for administration to the animals.

### 2.2. Animals and Treatments

Eight-week-old C57BL/6J mice (9 females and 5 males) were obtained from the Laboratory Animal Center of Southern Medical University. All animal experiments were conducted in accordance with the National Institutes of Health guidelines outlined in the Guide for the Care and Use of Laboratory Animals and were approved by the Experimental Animal Ethics Review Committee of Southern Medical University. All animal experiments, data reporting, and statistical analyses adhered to the ARRIVE guidelines (Supplementary Material S1) [23,24,25,26,27,28]. The animals were housed under specific-pathogen-free (SPF) conditions with controlled temperature (20–25 °C) and humidity (40–60%) under a 12 h light/dark cycle. Food and water were provided ad libitum.

After a one-week acclimation period, female mice were mated with males at a ratio of 1:1 to 2:1 in the same cage. Upon detection of sperm-positive vaginal smears, females were transferred to individual cages, and this day was designated as gestational day 0 (GD0). Pregnant mice were randomly assigned to three groups (*n* = 3 per group): control (Milli-Q water), PFOA (1 mg/kg body weight/day), and GenX (2 mg/kg body weight/day). Treatments were administered via oral gavage daily from GD0 to parturition. The dosage selection was based on regulatory guidelines. The U.S. Environmental Protection Agency (EPA) established a drinking water lifetime health advisory level (HAL) of 70 ppt for PFOA, on the basis of a developmental toxicity study in mice that identified 1 mg/kg/day as the lowest observed adverse effect level (LOAEL) [29,30]. Accordingly, dosages of 1 mg/kg/day for PFOA were selected. The dosage of GenX was chosen primarily on the basis of the findings from our preliminary study [21], as well as observations from previous research on adverse maternal and placental outcomes, in which the LOAEL for GenX exposure was identified as 2 mg/kg/day [31]. Additionally, the provisional health goal for GenX in North Carolina is set at 140 ppt, which is twice that of the PFOA HAL [31]. Accordingly, a dosage of 2 mg/kg/day for GenX was selected.

The dams were anesthetized with 60 mg/kg pentobarbital immediately after parturition. Following anesthesia, cardiac puncture was performed to collect blood, and the heart was then thoroughly perfused with saline. Liver tissues were subsequently collected promptly. The blood was centrifuged at 4 °C for 10 min to obtain the serum, which was stored at −80 °C for biochemical analysis. Liver tissue was divided into three portions: one for RNA sequencing analysis, another fixed in 4% paraformaldehyde solution for histopathological analysis, and the remainder flash-frozen in liquid nitrogen and stored at −80 °C for subsequent analyses.

### 2.3. Histopathological Analyses

Liver tissues were fixed in 4% paraformaldehyde solution for 24 h, followed by dehydration, clearing, and paraffin embedding. The embedded tissues were sectioned at a thickness of 3 μm and stained with hematoxylin and eosin (H&E). The stained sections were examined under an Olympus BX53 microscope (Olympus, Tokyo, Japan).

Histological assessment was conducted by three independent veterinary pathologists who were blinded to the treatment groups. In accordance with our previous studies [21,32], the evaluation of liver sections from pregnant mice followed standardized criteria, utilizing the Histological Activity Index (HAI) scoring system for intralobular hepatocyte degeneration, focal necrosis, and periportal inflammation combined with the NAFLD Activity Score (NAS) system for the assessment of steatosis.

### 2.4. Serum Biochemical Analyses

Serum alanine aminotransferase (ALT) and aspartate aminotransferase (AST) levels were measured via mouse-specific ELISA kits (Jiangsu Meibiao Biological Technology, Yancheng, China) following the manufacturer’s instructions.

### 2.5. RNA Sequencing and Bioinformatics Analyses

Total RNA was extracted from liver tissues via TRIzol^®^ Reagent following the manufacturer’s protocol. RNA quality and quantity were assessed via a 5300 Bioanalyzer and an ND-2000 Spectrophotometer, respectively. Only high-quality RNA samples (OD260/280 = 1.8–2.2, OD260/230 ≥ 2.0, RIN ≥ 6.5, 28S:18S ≥ 1.0, >1 μg) were used for library preparation. RNA purification, reverse transcription, library construction, and sequencing were performed by Majorbio Biopharm Biotechnology following Illumina protocols. The mRNA libraries were prepared via an Illumina Stranded mRNA Prep Kit with 1 μg of total RNA per sample. The libraries were sequenced on a NovaSeq 6000 platform with 2 × 150 bp paired-end reads.

The raw sequencing reads were quality controlled and trimmed via fastp, followed by alignment to the reference genome via HISAT2. StringTie was used for transcript assembly, and gene expression was quantified as transcripts per million (TPM) values. Differentially expressed genes (DEGs) were identified via either DESeq2 (|log2FC| ≥ 1 and FDR ≤ 0.05) or DEGseq (|log2FC| ≥ 1 and FDR ≤ 0.001). All bioinformatic analyses were performed on the Majorbio Cloud Platform.

### 2.6. Quantitative RT-PCR (RT-qPCR) Analysis

Total RNA was extracted from liver tissue via TRIzol reagent. The RNA concentration was measured via a NanoDrop 2000 Spectrophotometer (Thermo Scientific, Waltham, MA, USA). Total RNA (500 ng) was reverse transcribed into complementary DNA via PrimeScript™ RT Master Mix (RR036A; Takara, Shiga, Japan) following the manufacturer’s instructions. Gene expression was quantified via TB Green™ Premix Ex Taq™ GC (RR820A, Takara, Shiga, Japan) on a Roche Light Cycler^®^ 96 system (Roche Life Science, Welwyn Garden, Herts, UK). Relative gene expression was calculated via the 2^−ΔΔCt^ method, with β-actin serving as the internal reference gene. The sequences of primers used in this study are listed in Supplementary Material S2.

### 2.7. Western Blotting Analysis

Liver tissue samples were homogenized in RIPA lysis buffer (WB-0072, Beijing Dingguo Changsheng Biotechnology Co., Ltd., Beijing, China) containing 1% phenylmethylsulfonyl fluoride (PMSF; WB-0072, Beijing Dingguo Changsheng Biotechnology Co., Ltd., Beijing, China). The homogenates were centrifuged at 12,000 rpm for 10 min at 4 °C, and the protein concentrations in the supernatants were determined via a BCA protein assay kit (Thermo Scientific, Waltham, MA, USA).

Equal amounts of protein from each sample were separated via sodium dodecyl sulfate–polyacrylamide gel electrophoresis (SDS–PAGE) and transferred onto polyvinylidene difluoride (PVDF) membranes (1620177, Bio-Rad Laboratories, Hercules, CA, USA). The membranes were incubated with primary antibodies overnight at 4 °C with gentle shaking, followed by incubation with appropriate horseradish peroxidase (HRP)-conjugated secondary antibodies for 1 h at room temperature. Protein bands were visualized using Meilunbio^®^ fg supersensitive ECL luminescence reagent (Meilunbio, Dalian, China) and a ChemiDoc MP Imaging System (Bio-Rad Laboratories, Hercules, CA, USA).

Band intensities were quantified via ImageJ software (version 1.8.0, National Institutes of Health, Bethesda, MD, USA), and the results were normalized to those of β-actin, which served as an internal loading control. The primary antibodies used were as follows: CYP4A1/A2/A3 (sc-53247, Santa Cruz, CA, USA), EGFR (sc-373746, Santa Cruz, CA, USA), c-Myc (sc-40, Santa Cruz, CA, USA), Oatp2 (sc-376424, Santa Cruz, CA, USA), β-catenin (sc-7963, Santa Cruz, CA, USA), TNFα (sc-52746, Santa Cruz, CA, USA), IL-6 (sc-57315, Santa Cruz, CA, USA), TGFβ1 (sc-130348, Santa Cruz, CA, USA), NLRP3 (T55651S, Abmart, Shanghai, China), GSDMD (AF4012, Affinity, PA, USA), caspase-1 (sc-398715, Santa Cruz, CA, USA), IL-18 (A16737, ABclonal, Wuhan, China), IL-1β (sc-52012, Santa Cruz, CA, USA), cleaved PARP-1 (sc-56196, Santa Cruz, CA, USA), p21 (sc-6246, Santa Cruz, CA, USA), p16 (YT3493, ImmunoWay, TX, USA), VCAM-1 (YT7839, ImmunoWay, TX, USA), ICAM-1 (YT2269, ImmunoWay, TX, USA), and β-Actin (sc-47778, Santa Cruz, CA, USA). The secondary antibodies used were mouse IgGκ BP-HRP (sc-516102, Santa Cruz, CA, USA) and mouse anti-rabbit IgG-HRP (sc-2357, Santa Cruz, CA, USA).

### 2.8. Statistical Analysis

The data are presented as the means ± standard deviations (SDs). One-way analysis of variance (ANOVA) was used to compare body weight, liver weight, liver-to-body weight ratio, serum ALT and AST levels, and histopathological scores among the three experimental groups. For RT–qPCR and Western blotting analyses, independent-samples t tests were used to assess differences between the two groups. Linear regression analysis was performed to evaluate the correlation between gene expression levels obtained from RNA sequencing and those obtained via RT–qPCR. All the statistical analyses were conducted via SPSS statistical software (version 25.0, IBM Corp., Armonk, NY, USA). A *p*-value < 0.05 was considered statistically significant.

## 3. Results

### 3.1. Gestational Exposure to PFOA or GenX Led to Changes in the Livers of Maternal Mice

**Figure 1:**
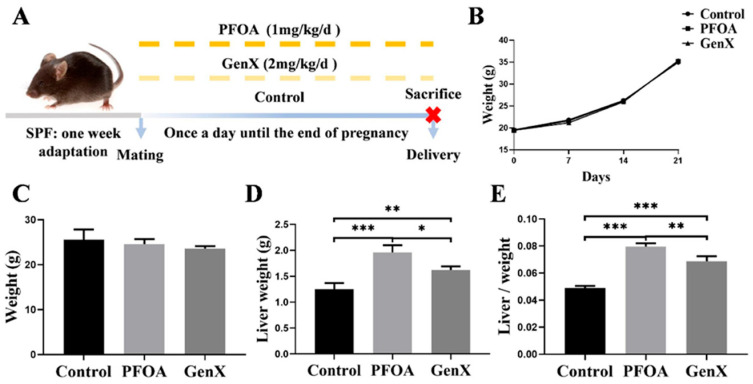
General observation of pregnant mice. (**A**) Schematic diagram of the experimental procedure. (**B**) Pregnancy weight change. (**C**) Final weights of the maternal mice after the experiment. (**D**) Liver weight. (**E**) Liver/weight ratio. *n* = 3 per group. * *p* < 0.05, ** *p* < 0.01, *** *p* < 0.001.

As shown in Figure 1A, all pregnant mice survived until the end of the experiment. No significant differences in gestational weight changes or final body weights were observed among the control, PFOA, and GenX groups (Figure 1B,C). However, compared with the control group, both the PFOA and GenX groups presented significantly increased liver weights and relative liver weights. Notably, the liver weights and relative liver weights in the GenX group were lower than those in the PFOA group (Figure 1D,E).

**Figure 2:**
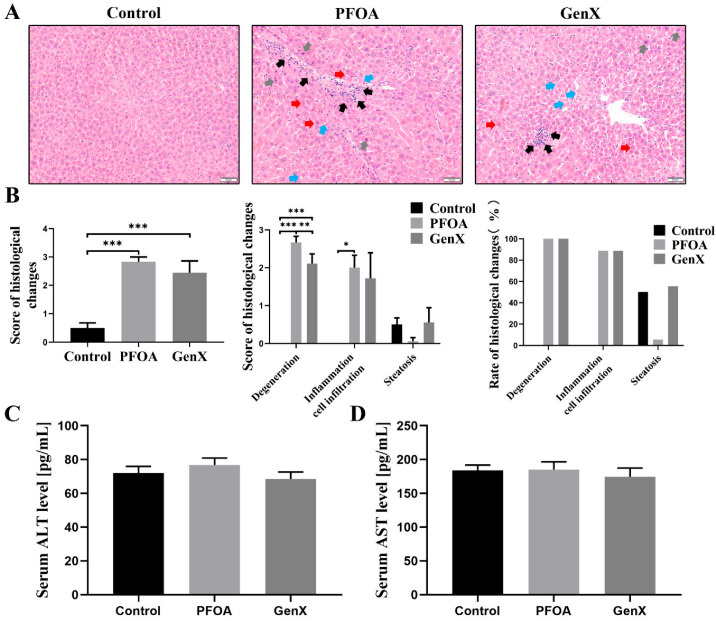
Histopathological features of liver tissues and serum levels of ALT and AST in pregnant mice. (**A**) Histopathological changes observed in maternal mice from the PFOA and GenX groups by H&E staining. Eosinophilic degeneration (red arrows), karyopyknosis (gray arrows), lipid vacuolation (blue arrows), and inflammatory infiltration (black arrows) were detected. Bar = 50 μm. (**B**) Histopathological scores of the maternal liver. (**C**) Serum ALT levels. (**D**) Serum AST levels. *n* = 3 per group. * *p* < 0.05, ** *p* < 0.01, *** *p* < 0.001.

Histopathological examination revealed no obvious pathological changes in liver tissues from the control group. In contrast, distinct histopathological alterations, including hepatocyte swelling, cellular disarrangement, eosinophilic degeneration (red arrows), karyopyknosis (gray arrows), lipid vacuolation (blue arrows), and increased inflammatory cell infiltration (black arrows), were observed in both the PFOA and GenX groups (Figure 2A). Liver histopathological scores, which reflect hepatocyte degeneration, inflammatory cell infiltration, and steatosis, were significantly greater in both the PFOA and GenX groups than in the control group (Figure 2B). Despite these histological changes, no significant differences in the serum ALT and AST levels were observed among the three groups (Figure 2C,D). These findings suggested that gestational exposure to either PFOA or GenX might induce hepatic histopathological changes in maternal mice, even in the absence of elevated serum transaminases.

### 3.2. Gestational GenX Exposure Caused Maternal Hepatic Injury by Disrupting Lipid and Bile Acid Metabolism

**Figure 3:**
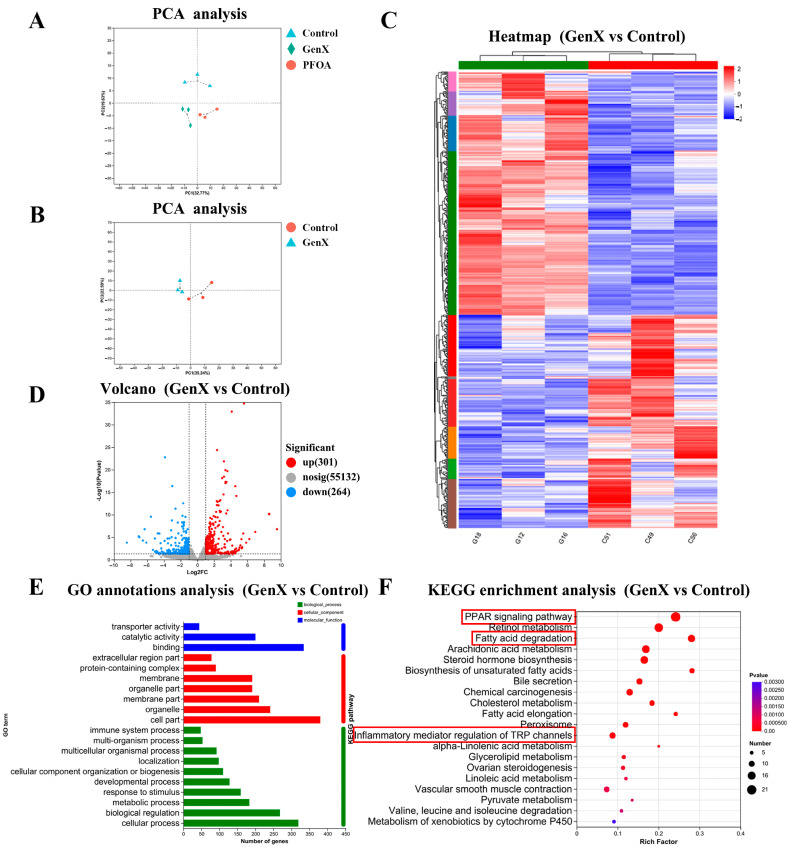
Transcriptome analysis of liver tissues from pregnant mice after GenX exposure. (**A**,**B**) Principal component analysis of maternal data. (**C**) Heatmap of differentially expressed genes (DEGs) between the control and GenX groups. (**D**) Volcano plot comparing gene expression between the two groups. (**E**) Gene Ontology (GO) annotation analysis comparing the control and GenX groups. (**F**) KEGG pathway enrichment analysis of DEGs in the control and GenX groups. *n* = 3 per group.

Principal component analysis (PCA) demonstrated clear separation between samples from the control, GenX, and PFOA groups, indicating significant transcriptomic differences among these groups (Figure 3A,B). Hierarchical cluster analysis visualized as a heatmap further revealed distinct mRNA expression patterns between the control and GenX groups (Figure 3C). Using the threshold criteria of *p* < 0.05 and a fold change ≥ 2, we identified 565 differentially expressed genes (DEGs) in the GenX group compared with the control group, with 264 downregulated and 301 upregulated genes (Figure 3D). These DEGs were categorized into functional groups through Gene Ontology (GO) annotation to predict their biological functions across biological processes, cellular components, and molecular functions (Figure 3E). Kyoto Encyclopedia of Genes and Genomes (KEGG) pathway enrichment analysis revealed that the DEGs in the GenX group were significantly enriched in 35 signaling pathways, including the PPAR signaling pathway, fatty acid degradation, arachidonic acid metabolism, biosynthesis of unsaturated fatty acids, bile secretion, cholesterol metabolism, inflammatory mediator regulation of TRP channels, glycerolipid metabolism, and the TGF-β signaling pathway (Figure 3F).

**Figure 4:**
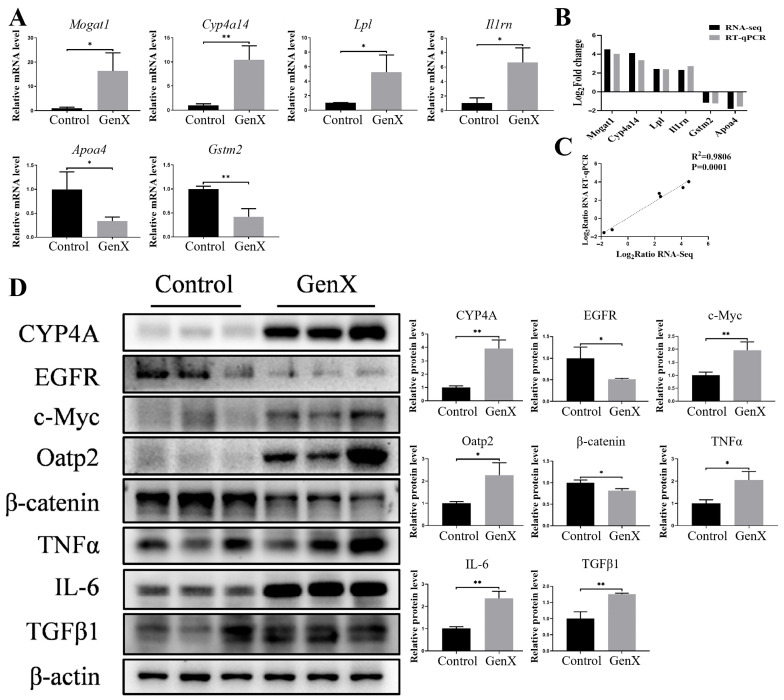
Analysis of mRNA and protein expression in the liver tissues of pregnant mice after GenX exposure. (**A**) mRNA expression levels in liver tissues of mice in the control and GenX groups. (**B**,**C**) Linear regression analysis of the RNA sequencing results and RT–qPCR results for the control and GenX groups. (**D**) Protein expression levels of specific genes in maternal liver from the GenX group determined by Western blot. *n* = 3 per group. * *p* < 0.05, ** *p* < 0.01.

To validate the RNA sequencing results, we randomly selected six genes (four upregulated genes and two downregulated genes) for RT–qPCR analysis. Consistent with the transcriptomic data, the mRNA levels of Mogat1, Cyp4a14, Lpl, and Il1rn were significantly increased, whereas those of Apoa4 and Gstm2 were significantly decreased in the GenX group compared with those in the control group (Figure 4A). Linear regression analysis revealed a strong correlation between the fold changes detected by RT–qPCR and RNA sequencing for these genes, confirming the reliability of the transcriptomic data (Figure 4B,C).

On the basis of the pathway enrichment results, we further investigated the expression of proteins related to lipid and bile acid metabolism. Compared with the control, GenX treatment significantly increased the protein expression of CYP4A and decreased the protein expression of EGFR, suggesting disruption of hepatic lipid metabolism. In the bile acid metabolism pathway, GenX exposure significantly increased c-Myc and Oatp2 protein levels while decreasing β-catenin expression, indicating the dysregulation of bile acid homeostasis. In addition, GenX exposure significantly upregulated the inflammatory markers TNFα and IL-6. The overexpression of the TGFβ1 protein in the GenX group suggested a compensatory response to the elevated expression of inflammatory factors (Figure 4D). Collectively, these results suggest that gestational GenX exposure induces hepatotoxicity primarily through the disruption of lipid and bile acid metabolism, leading to a cascade-mediated inflammatory response.

### 3.3. Gestational PFOA Exposure Induced Maternal Hepatotoxicity by Activating Pyroptosis

**Figure 5:**
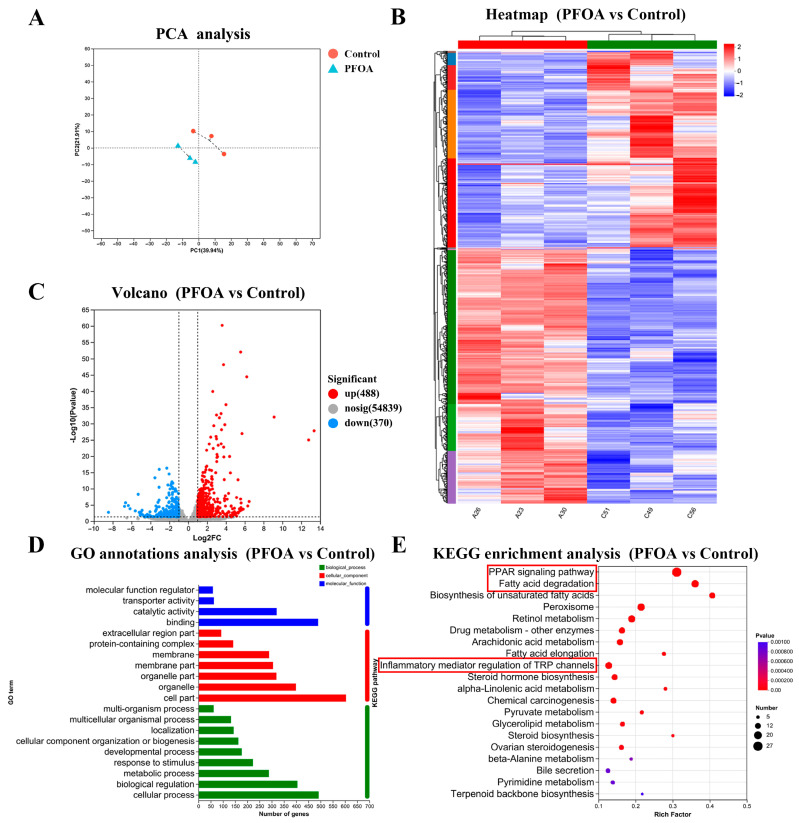
Transcriptome analysis of liver tissues from pregnant mice after PFOA exposure. (**A**) Principal component analysis of maternal mice. (**B**) Heatmap of DEGs between the control and PFOA groups. (**C**) Volcano plot comparing gene expression between the two groups. (**D**) GO annotation analysis comparing the control and PFOA groups. (**E**) KEGG pathway enrichment analysis of DEGs in the control and PFOA groups. *n* = 3 per group.

PCA of RNA sequencing data from maternal liver samples revealed clear separation between the control and PFOA groups, indicating distinct transcriptomic profiles (Figure 5A). This distinction was further visualized in the hierarchical cluster analysis heatmap, which revealed significant differences in the mRNA expression patterns between the two groups (Figure 5B). Using the criteria of *p* < 0.05 and a fold change ≥ 2, we identified 858 DEGs in the PFOA group compared with the control group, with 370 downregulated and 488 upregulated genes, as illustrated in the volcano plot (Figure 5C). These DEGs were functionally categorized through GO annotation analysis (Figure 5D). According to the KEGG pathway enrichment analysis, the DEGs were enriched in 58 significant pathways in the PFOA group, including the PPAR signaling pathway, fatty acid degradation, retinol metabolism, arachidonic acid metabolism, inflammatory mediator regulation of TRP channels, cellular senescence, and cell adhesion molecules (Figure 5E).

**Figure 6:**
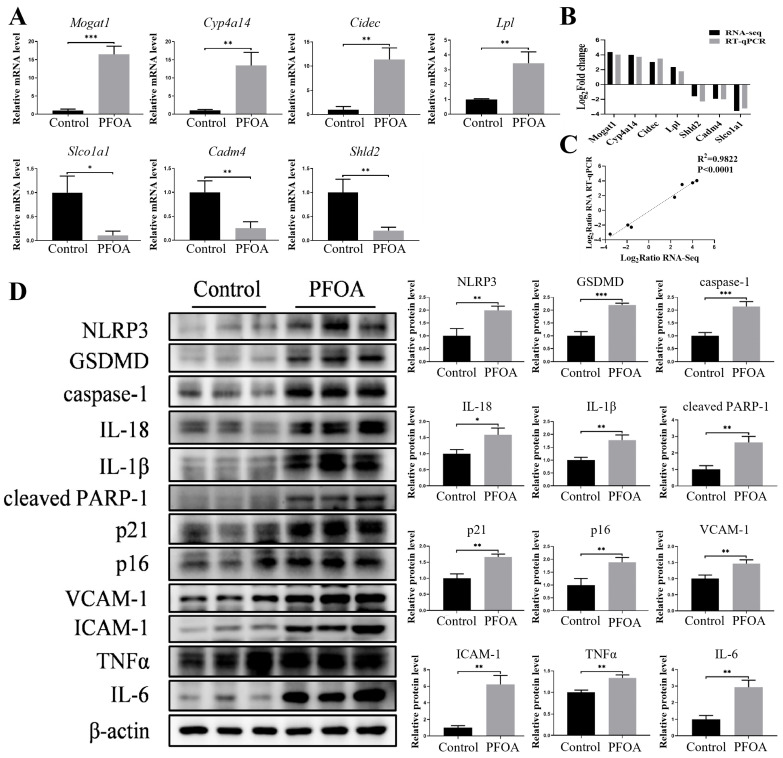
Analysis of mRNA and protein expression in the liver tissues of pregnant mice after PFOA exposure. (**A**) mRNA expression levels in liver tissues of mice in the control and PFOA groups. (**B**,**C**) Linear regression analysis of the RNA sequencing results and RT–qPCR results for the control and PFOA groups. (**D**) Protein expression levels of specific genes in maternal livers from the PFOA group determined by Western blot. *n* = 3 per group. * *p* < 0.05, ** *p* < 0.01, *** *p* < 0.001.

To validate the RNA sequencing results, we randomly selected seven DEGs for RT–qPCR analysis, including four upregulated genes (Mogat1, Cyp4a14, Cidec, and Lpl) and three downregulated genes (Slco1a1, Cadm4, and Shld2) (Figure 6A). Because multiple pathways were enriched in both the PFOA and GenX groups, the three genes Mogat1, Cyp4a14, and Lpl were included in the genetic verification for both groups. Linear regression analysis revealed a strong correlation between the fold changes measured via RT–qPCR and those measured via RNA sequencing, confirming the reliability of our transcriptomic data (Figure 6B,C).

According to the KEGG enrichment analysis, the PPAR signaling pathway, which has been reported to promote pyroptosis, was significantly enriched at the top [33]. Pyroptosis contributes to PFAS-induced damage to multiple organs, including the liver, lungs, and immune system [34,35,36]. Western blot analysis revealed that PFOA treatment significantly upregulated the expression of pyroptosis-related proteins, including NLRP3, GSDMD, caspase-1, IL-18, and IL-1β, indicating the induction of pyroptosis in the maternal liver. The expression of cleaved PARP-1, p21, p16, and the cell adhesion molecules VCAM-1 and ICAM-1 was significantly increased by PFOA exposure. Consistent with pyroptosis induction, the overexpression of TNFα and IL-6 was also observed, suggesting the release of inflammatory factors in the maternal liver (Figure 6D). Collectively, these results indicated that gestational PFOA exposure might induce maternal hepatotoxicity through pyroptosis induction.

## 4. Discussion

In the present study, gestational exposure to either GenX or PFOA resulted in significant increases in both absolute and relative liver weights in maternal mice. Furthermore, both exposure groups presented significant histopathological alterations in the liver. These findings align with those of previous studies demonstrating that prenatal exposure to GenX or PFOA can induce hepatic injury in maternal mice [37,38,39,40]. Interestingly, despite the observed histopathological changes, we did not detect elevated serum ALT and AST levels, which contrasts with findings from several other studies [21,41,42,43]. This discrepancy might be attributed to the relatively short exposure period, which was limited to the gestational period. Nevertheless, the histopathological findings provided compelling evidence that gestational exposure to GenX or PFOA induced hepatic injury in maternal mice, even in the absence of elevated serum transaminases.

To elucidate the molecular mechanisms involved, we performed a comprehensive transcriptomic analysis of maternal liver tissues. KEGG pathway enrichment analyses revealed that DEGs in the GenX group were significantly enriched in 35 pathways, including the PPAR signaling pathway, fatty acid degradation, inflammatory mediator regulation of TRP channels, and the TGF-β signaling pathway. Interestingly, the 58 pathways enriched in the PFOA group overlapped with those enriched in the GenX group, including the PPAR signaling pathway, retinol metabolism, fatty acid degradation, arachidonic acid metabolism, biosynthesis of unsaturated fatty acids, bile secretion, and inflammatory mediator regulation of TRP channels. These findings suggested that the compounds might induce hepatic injury through both shared and distinct mechanisms, which is consistent with our previous findings [21].

Activation of the PPAR signaling pathway is contributing to PFAS-induced immunotoxicity and cellular differentiation processes [44,45]. PFOA has been shown to activate PPARα during pregnancy in mice, affecting developmental outcomes through alterations in gene expression related to lipid and glucose homeostasis [46]. Similarly, GenX analogs have been reported to induce hepatotoxicity primarily through PPARα activation, with some analogs exhibiting greater toxicity than GenX itself [47]. Even at low doses, both PFOA and GenX can disrupt hepatic lipid metabolism via the PPARα signaling pathway, promoting steatosis and altering the liver transcriptome [48].

In the GenX group, multiple metabolic pathways were enriched, suggesting that disruption of lipid and bile acid metabolism is a primary mechanism of GenX-induced hepatotoxicity during pregnancy. Building on our previous research linking metabolic disruption to hepatotoxicity [32], we conducted targeted investigations of these metabolic pathways. Protein expression analyses confirmed that gestational GenX exposure significantly altered the expression of key regulators of lipid metabolism (increased CYP4A and decreased EGFR) and bile acid homeostasis (increased c-Myc and Oatp2 and decreased β-catenin). These findings are consistent with studies in male mice showing that GenX and its novel analogs disrupt bile acid metabolism [49] and fatty acid metabolism [50]. Notably, even at environmentally relevant concentrations in drinking water, GenX has been shown to interfere with hepatic lipid metabolism through the PPARα signaling pathway, leading to metabolic dysfunction and liver injury [22]. Additionally, the overexpression of TGFβ1 might compensate for the increase in the levels of the inflammatory factors TNFα and IL-6 in the livers of the GenX group. The release of inflammatory cytokines is closely related to metabolic disorders [51]. Collectively, our findings provide further evidence that GenX exposure during pregnancy might perturb hepatic lipid and bile acid metabolism, leading to inflammatory responses and hepatic injury.

The role of pyroptosis has been highlighted in previous studies on PFAS-induced injury in multiple organs, including the liver [35], lungs [34], and immune system [36]. In addition, hepatic steatosis progression is accompanied by pyroptosis and chronic inflammation in patients with metabolic-associated fatty liver disease (MAFLD) and in obese mouse models [52]. On the basis of the KEGG enrichment analysis, the PPAR signaling pathway was enriched at the top in the PFOA group, which has been reported to promote pyroptosis [33]. Our results confirmed that gestational PFOA exposure significantly upregulated the expression of pyroptosis mediators, including NLRP3, GSDMD, caspase-1, IL-18, IL-1β, TNFα, and IL-6. In this context, the overexpression of cell adhesion molecules (VCAM-1, ICAM-1) might facilitate leukocyte recruitment and migration during inflammatory responses [53] and promote the release of inflammatory mediators. On the basis of these findings, we propose a mechanistic model whereby gestational PFOA exposure triggers hepatic injury through the activation of pyroptosis in hepatocytes, accompanied by apoptosis and increased expression of cell adhesion molecules.

In conclusion, our study demonstrated that gestational exposure to either GenX or PFOA induced hepatic injury in maternal mice, albeit through distinct molecular mechanisms. GenX predominantly disrupted lipid and bile acid metabolism, whereas PFOA primarily triggered pyroptosis and enhanced cellular adhesion. Despite these mechanistic differences, both compounds ultimately stimulated the release of inflammatory mediators. These findings provide novel insights into the hepatotoxic mechanisms of PFOA and its replacement, GenX, during pregnancy. This study has several limitations that warrant further investigation. The U.S. EPA recently proposed a new HAL for GenX in drinking water at 10 ppt [54], which is considerably lower than the exposure levels in our study. Therefore, future research should examine the effects of these compounds at environmentally relevant concentrations, particularly around regulatory HALs. The low number of mice per group might limit the statistical power of this study, affect the ability to detect significant differences between groups, and increase the risk of Type II errors (failing to reject a false null hypothesis). Hence, studies with larger sample sizes and longer follow-up periods would help elucidate the long-term consequences of gestational PFAS exposure for both maternal and offspring health. Such research is critical for comprehensive risk assessment and the development of effective public health policies related to these persistent environmental contaminants.

## Data Availability

Data will be made available on request.

## Supplementary Materials

The following supporting information can be downloaded at https://www.mdpi.com/article/10.3390/toxics13080617/s1, Supplementary Material S1: The ARRIVE Essential 10: Compliance Questionnaire; Supplementary Material S2: Primers for RT-qPCR analysis [23,24,25,26,27,28].
