# Supplementary material for: Gestational GenX Exposure Induces Maternal Hepatotoxicity by Disrupting the Lipid and Bile Acid Metabolism Distinguished from PFOA-Induced Pyroptosis
Source: Toxics. 2025 Jul 24;13(8):617. doi: 10.3390/toxics13080617 (PMC12389976; doi:10.3390/toxics13080617)
Supplement: Supplementary file 1 [file toxics-13-00617-s001.zip › Supplementary material S2_Primers for RT-qPCR analysis.pdf]

Table S1

Primers for RT-qPCR analysis.

Gene name	Forward (5′-3′)	Reverse (5′-3′)
Mogat1	CTCGTGCAGGTGTGCATTG	GCGTTTTGACAAGACAGATTGG
Cyp4a14	TCTGGGTTCTTCCAATGGGC	GGACTCGTATATTGCTCCCCG
Lpl	TTGCCCTAAGGACCCCTGAA	TTGAAGTGGCAGTTAGACACAG
Il1rn	TAGACATGGTGCCTATTGACCT	TCGTGACTATAAGGGGCTCTTC
Apoa4	CCAATGTGGTGTGGGATTACTT	AGTGACATCCGTCTTCTGAAAC
Gstm2	ACACCCGCATACAGTTGGC	TGCTTGCCCAGAAACTCAGAG
Cidec	ATGGACTACGCCATGAAGTCT	CGGTGCTAACACGACAGGG
Slco1a1	ACTCCCATAATGCCCTTGG	TAATCGGGCCAACAATCTTC
Cadm4	ACAGGAAGTACAGACCGAGAAT	ATGACGACTATGGACCCATCG
Shld2	CCACTGAAAACGACAGTATCACA	TTGGGGACTTGACAGTCTTCC
